# Rehabilitation Professionals' Self‐Perceived Competence in Safe Patient Handling and Mobility Methods Before and After Training: A Cohort Study

**DOI:** 10.1002/hsr2.70755

**Published:** 2025-04-23

**Authors:** Jeanette Melin, Nicola Parmelund, Magnus Johansson

**Affiliations:** ^1^ Department of Health and Caring Sciences Linneaus University Kalmar Sweden; ^2^ Department of Leadership, Demand and Control Swedish Defence University Karlstad Sweden; ^3^ Institute of Social Sciences Södertörn University Stockholm Sweden; ^4^ HMC Hjälpmedelcenter Sweden: A National Knowledge Center Gothenburg Sweden; ^5^ Division Built Environment, System Transition RISE Research Institutes of Sweden Stockholm Sweden; ^6^ Department of Clinical Neuroscience, Karolinska Institutet, & Stockholm Health Care Services Centre for Psychiatry Research Region Stockholm Sweden

**Keywords:** education program for patient handling, person‐centered care, safe patient handling and mobility, transfer techniques

## Abstract

**Background and Aim:**

To address knowledge gaps in safe patient handling and mobility methods (SPHMM) among rehabilitation professionals in Sweden, a national knowledge center, HMC, provides training on SPHMM. The study's aim was to report on outcomes at 3 months after training in terms of self‐perceived competence in SPHMM and self‐perceived utility of acquired competence in SPHMM.

**Methods:**

Training participants (occupational therapists and physiotherapists) completed a study‐specific questionnaire, with 1065 doing so at 3 weeks before the training and 389 at 3 months after training. Linear mixed models were used for analyses.

**Results:**

Self‐perceived competence improved significantly at 3 months after training (*p* < 0.001). Occupational therapists, specifically, and rehabilitation professionals generally with less clinical experience reported greater improvement compared to physiotherapists and those with longer experience (*p* < 0.001). Rehabilitation professionals overall with shorter clinical experience also reported higher self‐perceived utility of the acquired SPHMM competence (*p* < 0.001), but occupational therapists and physiotherapists did not differ significantly (*p* = 0.369).

**Conclusion:**

Among rehabilitation professionals, greater self‐perceived competence and self‐perceived utility of the acquired competence in SPHMM is possible after HMC training. Improvements were greater for those with shorter clinical experience. By enhancing competence among rehabilitation professionals, HMC training has the potential to advance working person‐centered and efficient transfer practices in interdisciplinary care settings.

## 1 Introduction

Patient handling and patient transfers are an integrated part of healthcare and nursing care. In hospitals, weight‐bearing activities for nursing care staff can reach as high as 1.8 tons each day [1], and in nursing homes, an assistant nurse may support over 90 transfers daily [2]. Historically, training efforts have primarily focused on improving the physical work environment and ensuring ergonomic safety for nursing care staff [3, 4, 5, 6, 7], emphasizing correct working positions and transfer techniques. However, despite these interventions, the anticipated benefits—such as reduced musculoskeletal injuries and improved patient handling practices—have not been fully realized [8, 9, 10], and risks persist for patients—especially older patients—and nursing care staff [6, 11, 12]. As a response, a comprehensive, person‐centered, and interdisciplinary approach to safe patient handling and mobility methods (SPHMM) is being encouraged [13, 14]. This is particularly relevant in Swedish municipal healthcare, where rehabilitation professionals play a key role in supporting nursing care staff in implementing SPHMM in day‐to‐day care. Thus, rehabilitation professionals—occupational therapists (OTs) and physiotherapists (PTs)—must be prepared for a wide range of patient handling and transfer situations, necessitating a solid foundation in SPHMM competence and skills. These can be effectively enhanced through structured SPHMM training.

In Sweden, the national knowledge center, HMC Sweden, has advocated for a person‐centered and round the clock SPHMM approach, emphasizing patients' fluctuating abilities, body functions, cognitive capacity, and environmental factors to promote active, safe, and gentle transfers [15]. A safety culture in patient handling depends on several critical factors, including work equipment, structured training, clear policies, effective risk assessment tools, and active employee engagement [16, 17, 18, 19, 20]. In person‐centered care, each person's unique reason, will, feelings, and needs must be acknowledged [21, 22, 23]. With respect to SPHMM, functional status, the physical environment (e.g., space, assistive devices), and the interaction between nursing care staff and patients also require careful consideration.

Despite the recognized importance of structured training and education in SPHMM for rehabilitation professionals, remains fragmented and inconsistently integrated into formal curricula. This may lead to variability in competency development and a lack of standardized training frameworks [24]. Although rehabilitation professionals play a pivotal role in overseeing SPHMM implementation and supporting nursing care staff in Sweden, their formal education provides little to no structured training in person‐centered patient handling and mobility methods. This lack of education may contribute to inconsistent application of SPHMM and limited capacity to support nursing care staff effectively. Without adequate competence, rehabilitation professionals may struggle to implement evidence‐based SPHMM, potentially impacting both caregiver safety and rehabilitation outcomes for older patients. To address this gap, HMC offers structured SPHMM programs for rehabilitation professionals grounded in person‐centered clinical reasoning and interdisciplinary collaboration and covers both manual and supported patient transfers with hoists and slings.

In general, training itself does not automatically result in changes to clinical routines. However, improved knowledge and skill levels may promote the efficient implementation of new working methods in the workplace. In line with self‐determination theory [25], some degree of transfer may be expected as a function of training in the context of more developed competence and skills [26]. Thus, the HMC training is not expected to ensure that SPHMM is implemented and maintained in practice but is rather aimed at improving competence among rehabilitation professionals. With improved competence, rehabilitation professionals may more effectively support and educate nursing care staff in applying SPHMM, thereby promoting person‐centered transfer practices in cross‐disciplinary teams around the clock.

To the best of our knowledge, few—if any—studies have explicitly examined SPHMM with a person‐centered framework, and no studies to date have specifically investigated HMC's training programs. This represents a significant gap in the literature and in clinical practice. Given that HMC training is designed to enhance competence among rehabilitation professionals, it is important to assess how they perceive their ability to apply SPHMM in clinical care. Self‐reports of competence provide insight not only into the theoretical understanding, but also into professionals' confidence in applying their knowledge in real‐world situations. Therefore, in the present study, we report on self‐perceived competence in SPHMM among rehabilitation professionals 3 months after HMC training, which we hypothesized would enhance self‐perceived SPHMM competence. We also explored possible differences between OTs and PTs and differences related to length of clinical experience. A secondary aim was to investigate the self‐perceived utility of the acquired SPHMM competence.

## 2 Methods

### 2.1 Study Design and Setting

To evaluate the outcome of HMC training on self‐perceived competence in SPHMM among rehabilitation professionals, we conducted a cohort study comparing self‐reports at 3 weeks before and 3 months after the HMC training based on the data registered by the company.

### 2.2 Participants

Since 2019, HMC has used a context‐specific questionnaire developed in‐house to evaluate SPHMM among rehabilitation professionals. The courses given during the period and included in this study lasted 3 or 4 days, were conducted in the municipalities or HMC facilities, and included either training on manual patient handling or transfers with hoists and slings for rehabilitation professionals. All participants enrolled for the SPHMM courses received an electronic link to the questionnaire 3 weeks before the course and 3 months after the course. The questionnaire was distributed via SurveyMonkey. All participants were informed that their responses could be used for research and development activities and that the data would be handled in accordance with the EU's General Data Protection Regulation.

For the present study, 1065 participants completed the questionnaire 3 weeks before the HMC training and 389 participants completed the questionnaire 3 months after the training. Table 1 shows respondents' professional characteristics and working experiences, which did not differ significantly among subgroups before or after training (profession *χ* ^2^ = 0.08, df = 1, *p* = 0.78; experience *χ* ^2^ = 2.36, df = 3, *p* = 0.50).

**Table 1:** Participant characteristics.

	Pre (*N* = 1065) *n* (%)	Post (*N* = 389) *n* (%)
Profession		
OT	572 (53.7)	214 (55.0)
PT	441 (41.4)	172 (44.2)
Missing	52 (4.9)	3 (0.8)
Experience		
≤ 2 years	357 (33.5)	125 (32.1)
2–5 years	300 (28.2)	122 (31.4)
5–10 years	162 (15.2)	62 (15.9)
≥ 10 years	230 (21.6)	73 (18.8)
Missing	16 (1.5)	7 (1.8)

Participants were asked to respond to questions about SPHMM and two demographic questions (profession and working experiences) and to provide e‐mails to allow for linking respondent outcomes before and after the training. Of the 389 respondents who completed the post‐training questionnaire, 206 (52.7%) could be linked to their pre‐training data through their e‐mail addresses. Their professions were distributed similarly to the full cohort (55.8% OT, 43.9% PT; 0.5% missing), but this subset had a higher proportion of professionals with shorter clinical experience and a lower proportion of professionals with the longest clinical experience (< 2 years: 37.9%; 2–5 years: 30.1%; 6–10 years: 16.0%; > 10 years: 15.5%; missing: 0.5%). Furthermore, a logistic regression model was conducted to assess whether experience, profession, or the measure at pre‐measurement affected the probability of responding at both time points; this showed no statistically significant results.

### 2.3 Education Program

HMC has provided SPHMM education for rehabilitation professionals since 1995. The education and practical training are underpinned by theory for early and progressive mobilization, aiming to ensure that the patient is as active as possible during transfers. This strategy is intended to promote perceived participation by using assistive devices matched to individual abilities and needs while simultaneously reducing the physical strain on nursing care staff. Over the years, the course content and numbers of courses have been continuous developed based on clinical experience and evolving best practice.

The HMC method is grounded in a theory of change, whereby organizations that implement it are expected to enhance SPHMM through a structured, three‐module framework. The first module focuses on the person, including a valid assessment of body functions, cognition, and the current activity status. The second module considers the physical environment in relation to the person's needs and abilities, typically involving the appropriate use of assistive devices. The third module addresses the care meeting, which involves a risk assessment and a collaborative evaluation based on the person's status before, during, and after a transfer.

A key feature is the structured use of profession‐specific “Transfer Assessment Protocols” (TAPs) [15], which serve not only as clinical reasoning tools but also as pedagogical supports for interdisciplinary communication and care planning. Rather than prioritizing staff safety alone, the HMC approach underscores the importance of balancing safety with mobility conservation—particularly in older patients—by ensuring assistive devices support, rather than replace, the person's abilities. This integrated focus on function, participation, and knowledge transfer positions the HMC method as a rehabilitation‐informed model for safe patient and handling, specifically adapted to the practical needs and interdisciplinary demands of everyday care settings.

Compared to other internationally established SPHMM frameworks [7, 27], (nya referensnr) the HMC method places greater emphasis on individualized functional assessment as the foundation for transfer planning, rather than starting with ergonomic principles, equipment use, or standardized movement techniques. While conventional frameworks typically focus on minimizing musculoskeletal risks through predefined equipment selection and procedural guidance, the HMC method integrates person‐centered reasoning, rehabilitation potential, and pedagogical strategies tailored to both rehabilitation professionals and nursing care staff.

A comprehensive description of the course content is available in Swedish at HMC. The training courses are typically conducted during working hours and employer funded.

### 2.4 Outcome Measures

Three weeks before the course and 3 months after the course, the participants were asked to respond to 12 questions about SPHMM (Table 2). In addition, 3 months after the course, an additional five items were included in the questionnaire. Some questions addressed the working process together with the care staff. These questions were not included in the current analysis focused on self‐perceived SPHMM competencies. Processes performed together with other care professionals represent another important dimension to explore but could not be expected to change in relation to the training unless all involved had completed the training.

**Table 2:** Questions used to measure self‐perceived competence in SPHMM (*n* = 7) and self‐perceived utility of the acquired SPHMM competence (*n* = 5), respectively.

		Question	Response options
Self‐perceived competence in SPHMM	1	Based on the patient's status, I know what to do, so that he or she can be active in his or her movement	Never = 0
			Seldom = 1
	2	I know what to do to support a transfer when the patient has reduced verbal ability	Sometimes = 2
			Often = 3
	3	I know how to teach safe and secure transfers to caring staff	Always = 4
	4	I know how the caring staff's own body weight can be used during transfers	
	5	I know how to do so that the transfers are not being too heavy for the caring staff	
	6	I know how to teach the caring staff to support a transfer where the patient has impaired cognitive ability	
	7	I know how to teach the caring staff to support a transfer where the patient has impaired verbal ability	
Self‐perceived utility of the acquired SPHMM competence	8	I find that the content of the training has been useful to me in my everyday work	Do not agree = 0
			Agree a little = 1
	9	I find that the training has changed the way I work	Agree somewhat = 2
			Mostly agree = 3
	10	I feel that I have a good/improved understanding and knowledge of different types of assistive devices after the training	Completely agree = 5
	11	I feel that my prescriptions are more appropriate after the training	
	12	I work more with specific rehabilitation in relation to transfers than I did before	

The outcome measure for self‐perceived competence in SPHMM is based on seven questions. Respondents were asked to rate each item on a five‐point Likert scale, with higher ratings indicating higher self‐perceived competence in SPHMM. The outcome measure of self‐perceived utility of the acquired SPHMM competence is based on five questions. Respondents were asked to rate each item on a five‐point Likert scale, with higher ratings indicating higher self‐perceived utility.

### 2.5 Data Analysis

Descriptive and statistical analyses were conducted in R version 4.4.3 [28]. For both outcome measures, Rasch analyses using the partial credit model with conditional maximum likelihood estimation were carried out using the easyRasch package [29] to obtain person measures for statistical analyses. The complete Rasch analyses and measurement properties are given in the Supporting Information S1: Appendix. Using person measures derived from a Rasch analysis has several advantages compared to using ordinal sum scores. Specifically, through the Rasch formula, observed ratings (i.e., ordinal data) are transformed and separated into measures of person and item attributes on a conjoint logit scale. Further details about Rasch analysis can be read elsewhere; for instance, see Andrich [30] and Cano and Hobart [31].

To evaluate differences in self‐perceived competence in SPHMM before and after HMC training for the full cohort and among subgroups, linear mixed models were applied using the lme4 package [32]. Models were estimated using restricted maximum likelihood and a nloptwrap optimizer. The use of maximum likelihood estimation makes it possible to include respondents with at least one measurement on the outcome variable, which results in maximal utilization of the data available.

Unique ID numbers were used for random effects (random intercepts) throughout the analyses, and the effects of profession (as a factor) and work experience (as an ordered factor) were modeled as fixed effects. Corresponding analyses also were conducted to evaluate group differences in self‐perceived utility of the acquired SPHMM competencies. All analyses and code are available in Supporting Information S1: Appendices. Models were compared using the Akaike information criterion, Bayesian information criterion, conditional and marginal *r* ^2^, and root mean square error. Model residuals were assessed using the R package “performance” [33] and since errors were found to be nonuniform we report bootstrapped standard errors using 500 simulations and the HC2 method for heteroscedasticity consistent standard errors [34]. The bootstrap was conducted using the R package “lmeresampler” [35]. Figures illustrating conditional effects were made using the R packages “marginaleffects” and all figures in the manuscript were generated using “ggplot2” [36, 37].

## 3 Results

### 3.1 Self‐Perceived Competence

**Figure 1:**
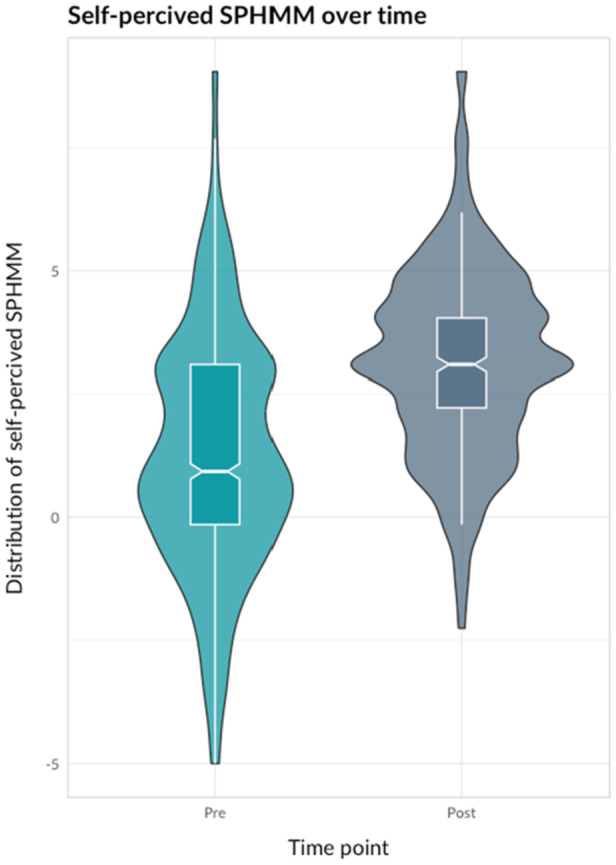
Violin plot comparing self‐perceived competence in SPHMM before and after HMC training for the full cohort.

**Figure 2:**
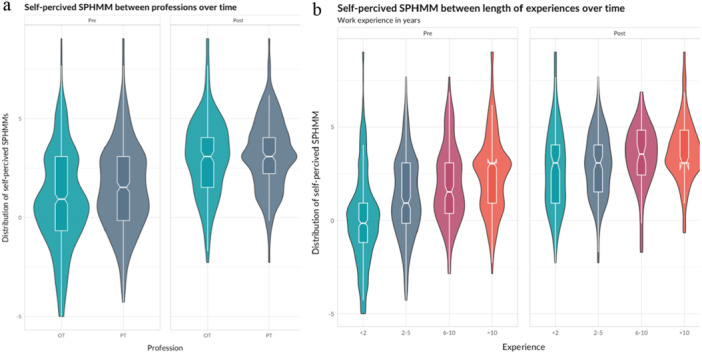
Violin and boxplots comparing self‐perceived competence in SPHMM before and after HMC training for (a) occupational therapists (OTs) and physiotherapists (PTs), and (b) rehabilitation professionals overall with different lengths of clinical experience.

The violin plot in Figure 1 shows the distribution of observed self‐perceived competence in SPHMM before and after HMC training for the full cohort. Corresponding plots in Figure 2 show comparisons of different professions and lengths of clinical experience before and after HMC training. As shown in the plots, the baseline average self‐perceived competence in SPHMM was significantly higher for PTs compared to OTs (*F* = 20.01, df = 1, *p* < 0.001) and for rehabilitation professionals with longer versus shorter clinical experience (*F* = 60.35, df = 3, *p* < 0.001).

As Figures 1 and 2a,b show, the median observed self‐perceived competence in SPHMM improved after the training. When self‐perceived competence in SPHMM was entered into a linear mixed model with individuals with unique ID numbers as a random effect, the fixed effect of time was statistically significant and positive (*β* = 1.95, 95% CI [1.76, 2.14], *t* [1447] = 17.65, *p* = 0.002). The explained variance of time for self‐perceived competence in SPHMM was substantial (conditional *R* ^2^ = 0.61), and the part related to the fixed effects was weak (marginal *R* ^2^ = 0.12).

**Figure 3:**
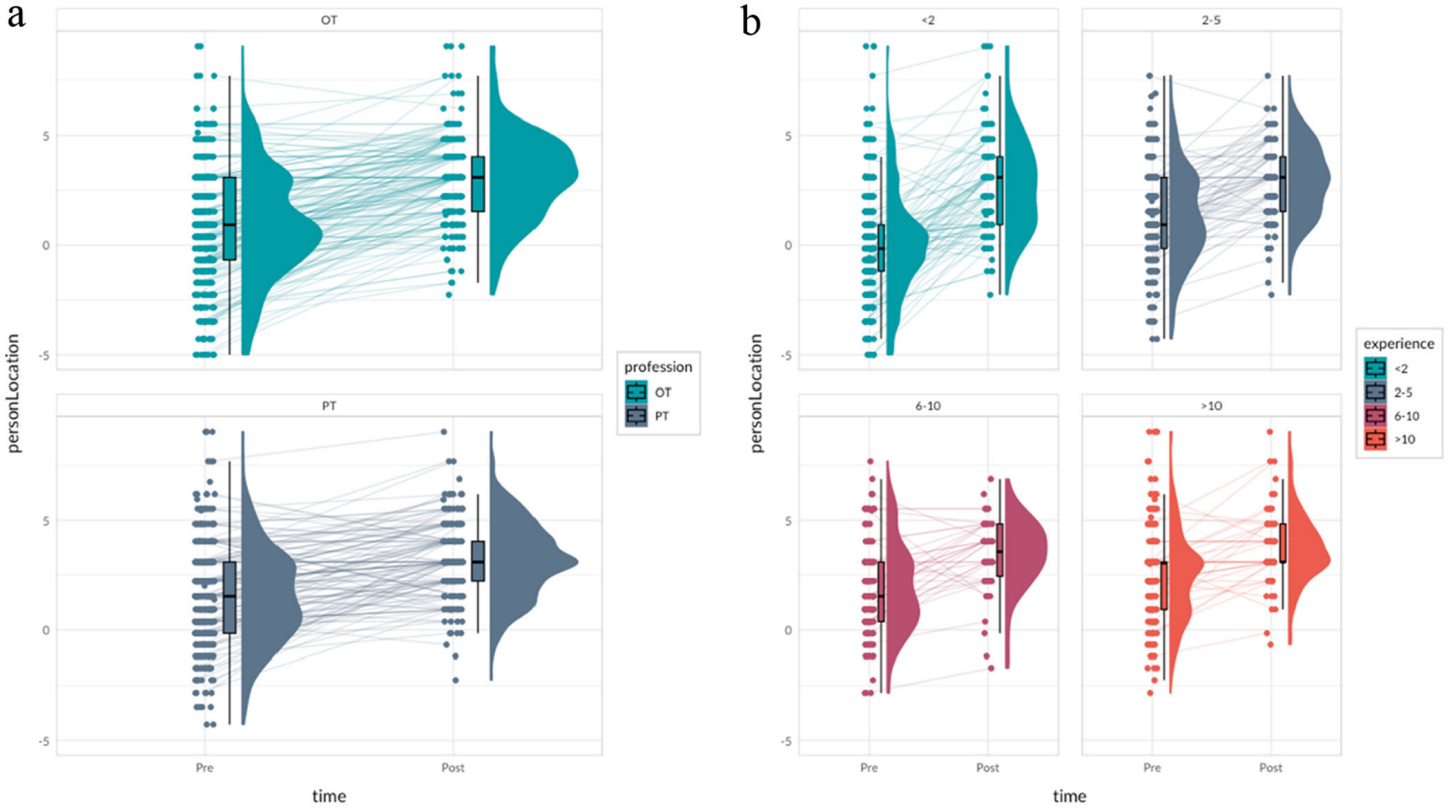
Raincloud plots showing observed self‐perceived competence in SPHMM before and after HMC training for (a) occupational therapists (OTs) and physiotherapists (PTs), and (b) rehabilitation professionals overall with different lengths of clinical experience. The raincloud plot combines the individual data points (swarm plot), a summary of the distribution (boxplot), and a smooth density curve to illustrate the overall data distribution.

**Figure 4:**
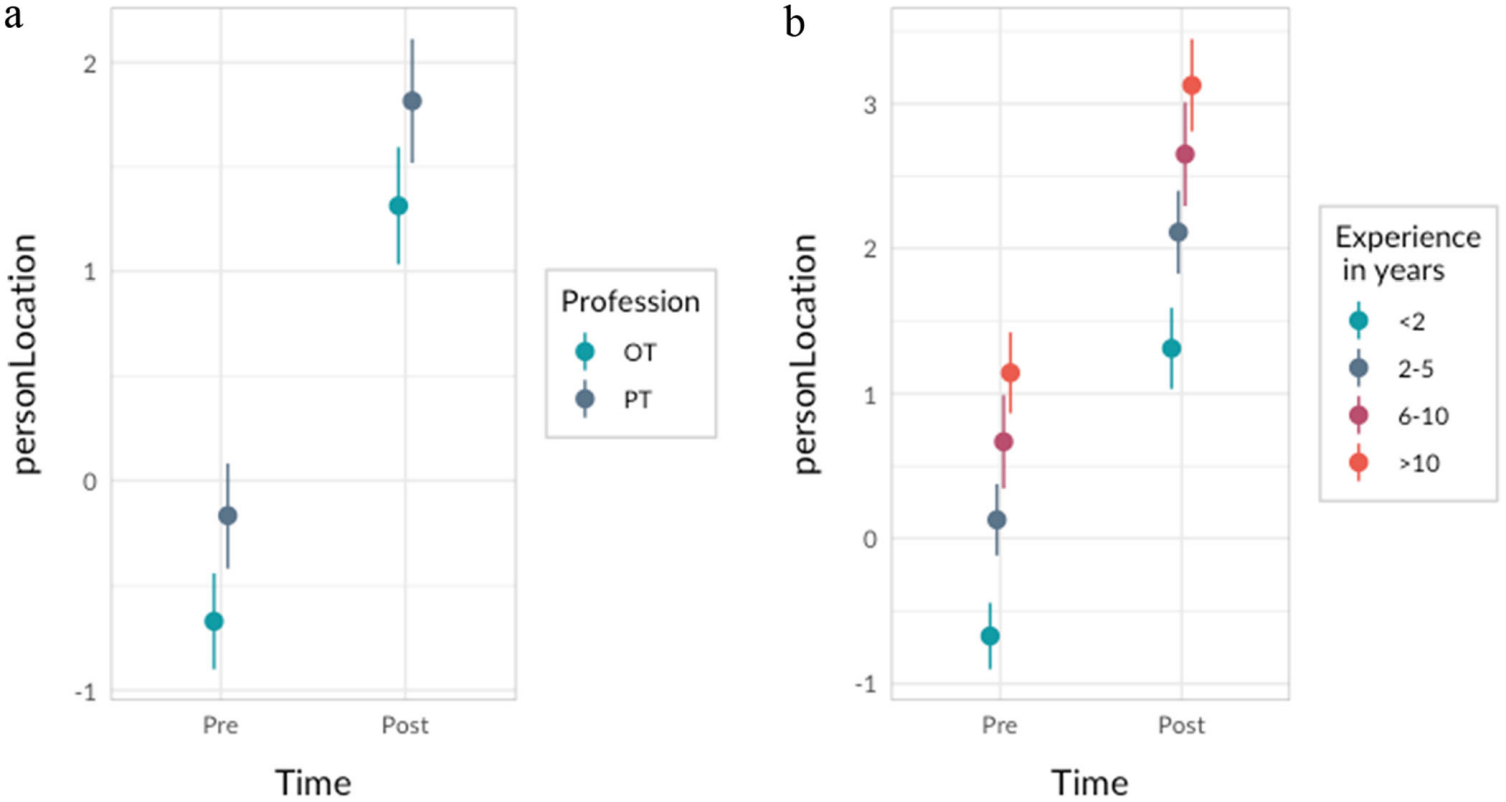
Conditional effects from the LMM for (a) occupational therapists (OTs) and physiotherapists (PTs), and (b) rehabilitation professionals overall with different lengths of clinical experience.

Raincloud plots in Figure 3a,b show the observed data for self‐perceived competence in SPHMM before and after training, by profession, and length of clinical experience. OTs specifically, and rehabilitation professionals in general with shorter clinical experience reported greater improvements. When unique ID numbers were retained as a random effect and profession and experience together with time were added as explanatory variables, the part related to the fixed effects increased from *R* ^2^ = 0.12 to 0.23 (see Table 3). Conditional effects from the LMM reported in Table 3 for profession and levels of experience are illustrated in Figure 4.

**Table 3:** Summary statistics of two linear mixed models.

	Person location ~ time + (1 | ID)	Person location ~ time + profession + experience + (1 | ID)
(Intercept)	1.24 (SE = 0.08, *p* = 0.002) 95% CI [1.10 to 1.40]	1.08 (SE = 0.09, *p* = 0.002) 95% CI [0.91 to 1.26]
Time	1.95 (SE = 0.10, *p* = 0.002) 95% CI [1.76 to 2.14]	1.98 (SE = 0.10, *p* = 0.002) 95% CI [1.78 to 2.17]
professionPT		0.50 (SE = 0.12, *p* = 0.002)95% CI [0.255 to 0.723]
Experience 2−5		1.34 (SE = 0.12, *p* = 0.002) 95% CI [0.255 to 0.723]
Experience 6−10		−0.16 (SE = 0.12, *p* = 0.204) 95% CI [−0.391 to 0.072]
Experience > 10		0.04 (SE = 0.13, *p* = 0.768) 95% CI [−0.185 to 0.269]
SD (Intercept ID)	1.75	1.42
SD (Observations)	1.56	1.58
Num.Obs.^a^	1451	1378
*r* ^2^ marginal	0.12	0.23
*r* ^2^ conditional	0.61	0.57
AIC	6494.0	5956.7
BIC	6515.1	5998.5
ICC	0.6	0.4
RMSE	1.12	1.23

Abbreviations: AIC = Akaike information criterion, BIC = Bayesian information criterion, CI = confidence interval, ICC = intra‐class correlation, ID = identification number, PT = physiotherapist, RMSE = restricted maximum likelihood estimation, SD = standard deviation, SE = standard error.

a The fewer number of observations in the right column is due to missing data on profession and/or experience.

Separate analyses of OTs and PTs are reported in Supporting Information S1: Appendix. With a corresponding linear mixed model with unique ID numbers as a random effect, the coefficient for time was statistically significant and positive in both groups (OT: *β* = 1.66, 95% CI [1.46, 1.87], *t* [782] = 15.70, *p* < 0.001; PT: *β* = 1.11, 95% CI [0.89, 1.33], *t* [609] = 19.98, *p* < 0.001). Differences were only minor if experience was included as a random effect in the total explained variance of the self‐perceived competence in SPHMM. As for the full cohort, the part related to fixed effects increased when experience was added as an explanatory variable together with time (for OTs, *R* ^2^ from 0.17 to 0.26; for PTs from 0.10 to 0.14). In all analyses, the explained variance was greater for OTs (*R* ^2^ 0.64−0.65) than for PTs (*R* ^2^ 0.56−0.57).

### 3.2 Self‐Perceived Utility of Acquired Competence

**Figure 5:**
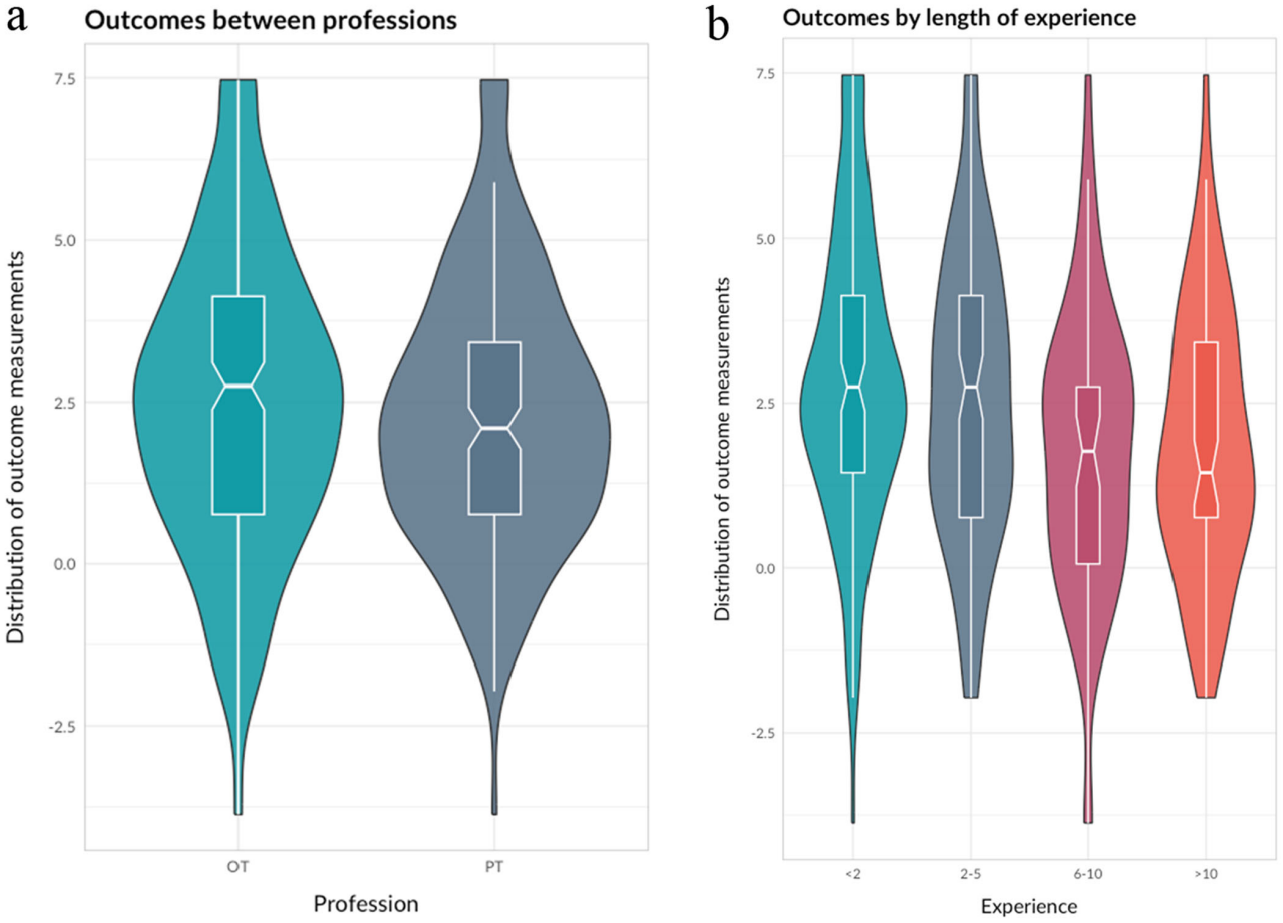
Violin plots comparing self‐perceived utility of the acquired SPHMM competence with different lengths of clinical experience for (a) occupational therapists (OTs) and physiotherapists (PTs), and (b) rehabilitation professionals overall.

The distribution of observed self‐perceived utility of the acquired SPHMM competence between professions and experiences is shown in Figure 5 and Supporting Information S1: Appendix. There was a statistically significant difference depending on the length of experience (*F* = 5.60, df = 3, *p* < 0.001); rehabilitation professionals overall with shorter clinical experience had higher self‐perceived utility of the acquired SPHMM competence. OTs and PTs did not differ significantly in terms of their self‐perceived utility of the acquired SPHMM competence (*F* = 0.81, df = 1, *p* = 0.369).

## 4 Discussion

The current results support the hypothesis that among rehabilitation professionals, enhanced self‐perceived competence in SPHMM can be achieved after HMC training. The findings also show greater improvements for OTs than for PTs and for professionals overall with shorter clinical experience. In addition, OTs and PTs did not differ in their self‐perceived utility of the acquired SPHMM competence, but overall, rehabilitation professionals with shorter clinical experience had a greater self‐perceived utility.

Baseline self‐perceived competence in SPHMM differed between OTs and PTs. This result is in keeping with previous findings among undergraduate OTs and PTs showing lower knowledge about safe patient handling among OTs [38, 39]. The HMC method starts with assessing body functions and cognition as an initial approach to person‐centered handling, which might explain the discrepancy between the two professional groups at baseline. PTs tend to have more experience in assessing body functions and structures, whereas OTs are more experienced in assessing activities [40, 41]. Despite a lower baseline average self‐perceived competence in SPHMM for OTs compared to PTs, however, average levels were about equal 3 months after completion of the HMC training. Respondents in the two groups also had similar self‐perceived utility of the acquired SPHMM competence 3 months after completing the HMC training.

It might be expected that professionals with shorter clinical experience would report greater improvements in self‐perceived competence in SPHMM and a higher utility of the acquired SPHMM ability. There is, indeed, little, if any, training in SPHMM for OT and PT undergraduates. Furthermore, it is possible that professionals with longer clinical experience underwent the SPHMM training with a more “reality‐based utility.” A lack of time and knowledge for proper assessment and clinical reasoning, availability of assistive devices, and competence within the interdisciplinary team in person‐centered patient handling could entail a lower self‐perceived utility after training for clinicians with more experience [39].

There is a call for more SPHMM training early in PT education to ensure enhanced clinical reasoning ability and increased competence in current SPHMM technology [42]. In line with a European review calling for standardization and establishing a framework for SPHMM [43], this approach may also be warranted in Sweden. Its implementation could better prepare undergraduates for their careers because SPHMM is prevalent in healthcare practice [24]. In addition, they could be more likely to advocate for SPHMM in clinical practice if didactic SPHMM training with assistive devices is incorporated early in their education [44]. Of note, this kind of training should rely not only on national or professional guidelines but also on an evidence‐based framework [43]. Guidance on how to integrate relevant content for educators is limited [42]. A person‐centered and interdisciplinary approach to SPHMM is presented in this study with HMC methodology that is theoretically based on a valid assessment of the patient's status and needs in the physical environment and a risk assessment of the patient handling, but further evidence is required on the outcome of HMC education programs in Sweden.

For all rehabilitation professionals, regardless of profession and length of clinical experience, the outcomes identified here suggest that many will still have room for improving self‐perceived competence in SPHMM. Specifically, as shown in Supporting Information S1: Appendices, the response option “often” was the most frequent, implying that further training might be needed to ensure that rehabilitation professionals know how to use appropriate knowledge and skills more regularly and in a broader variety of situations. HMC provides further training programs for advanced learning, including SPHMM, tailored for patients with obesity, pain, or dementia. With a longer time to follow‐up, the self‐perceived competence in SPHMM might have improved even more as the professionals built on the foundation of methodology from the HMC training by applying it when presented with new patient situations.

OTs and PTs are expected to work as rehabilitation consultants in Swedish municipalities. They assess rehabilitation needs for patients, but most rehabilitation occurs through the nursing care staff acting on a healthcare prescription. Within the interdisciplinary team, care staff also need competence in person‐centered patient handling, as well as knowledge about using assistive devices available at the workplace. Historically, education programs for SPHMM in Swedish municipalities are designed for nursing care staff and focused on favorable working positions and transfer techniques to ensure a safe physical work environment, rather than starting from a patient's abilities and resources to promote mobility optimization [5, 6, 10, 20].

Competence is associated with self‐determination theory [25], which positions the role of training in behavioral change. Autonomy, relatedness, and beneficence are also cornerstones in self‐determination theory, and all affect working life differently in diverse contexts and for a variety of functions [45, 46]. Two related theories of behavior change are the theory of planned behavior [47] and socio‐cognitive theory [48]. The conceptual overlap between competence and perceived behavioral control (a key component in the theory of planned behavior) is not clear [49]; however, competence and self‐efficacy (a key component in social‐cognitive theory), although related, are not equivalent [50]. It might be useful in these contexts to consider the HMC training that addresses these related abilities, especially as a person‐centered approach must tailor patient handling according to individual needs and situations. Professionals need a broad competence and confidence in transferring knowledge to new situations (i.e., perceive behavioral control and have self‐efficacy), as in clinical settings with variable patient abilities and needs and preconditions in the room (e.g., bed placement, compensatory devices).

In practical terms, HMC training can support rehabilitation professionals in strengthening their competence in SPHMM and increasing their effectiveness in training nursing care staff. Patient and occupational safety are closely intertwined and essential for achieving organizational goals [51], such as promoting active patient participation and minimizing the risk of work‐related strain injuries. When nursing care staff can engage patients and encourage them to use their capabilities during transfers, these daily care situations not only support the patient's physical and social functioning but also reduce the physical burden on nursing care staff. In turn, this may contribute to decreased work‐related injuries and lower sick leave rates among care personnel.

### 4.1 Methodological Considerations

A weakness of this study could be the use of a questionnaire developed in‐house for assessing self‐perceived competence in SPHMM and the self‐perceived utility of the acquired SPHMM competence. However, Rasch's analyses (Supporting Information S1: Appendices 1 and 2) of the data to assess measurement properties and produce interval‐level person measures for statistical analyses is a strength. To our knowledge, no similar questionnaire has been subjected to such assessments. We encourage others to build on the items used in this study to advance measurement quality further and contribute to a basis for shared understanding of self‐perceived SPHMM competence across training programs and studies. A further weakness in this study is the low response rate at the 3‐month follow‐up. The low response rate poses a risk of selection bias—as respondents may systematically differ from nonrespondents—and a nonresponse bias—perspectives from those who did not respond are missing—potentially reducing the statistical power and skewing the results. A proper drop‐out analysis was not possible to control for or implementation of SPHMM by OTs and PTs in everyday practice.

The choice of a 3‐month follow‐up period can be seen as somewhat arbitrary, as to the best of our knowledge, no previous studies with a similar design exist for direct comparison. Three months may be too short to capture sustained changes in competence and utility or, conversely, too long to detect immediate impacts that might diminish over time. The follow‐up period was, therefore, a pragmatic decision, balancing feasibility and the desire to assess both short‐term retention and potential early application of training content in clinical practice.

The present study opted for self‐perceived competence in SPHMM and self‐perceived utility of the acquired SPHMM competence rather than an objective knowledge‐based test. This decision was based on the recognition that such tests assess only partial knowledge by focusing on predefined cases, whereas SPHMM involves complex, context‐dependent decision‐making where multiple solutions may be valid. With the self‐reports used, we aimed to capture not only the theoretical breadth of competence but also how confident respondents feel in applying their knowledge in real‐world scenarios. However, future studies could complement this approach with patient case examinations to further explore practical application.

Furthermore, the lack of a control group and of randomized selection limits the interpretation of results as representing causal effects of the training, and the findings thus should be viewed with caution. Nevertheless, this initial study on the subject using existing data is a crucial first step in understanding the outcomes of SPHMM, even as caution should be applied when generalizing any improvements in self‐perceived competence in SPHMM and self‐perceived utility of the acquired SPHMM competence. We stress that more studies are needed with controlled designs to enhance the knowledge base.

The HMC training itself does not change working routines but offers skills and knowledge to efficiently forward change in working methods. Thus, the training is a key starting point that is intended to lead to further organizational change and support, including change advocates and support at all levels, from management to front‐line employees, to improve the working methods related to SPHMM [16]. As a part of this approach, HMC is currently running an implementation program together with a Swedish municipality. The aim is a better understanding of the kind of support an organization needs to ensure that rehabilitation professionals can transfer their knowledge to nursing care staff and, in turn, affect physical exertion during work and reduce costs associated with sick leave because of work‐related strain injuries.

## 5 Conclusion

The results of this study highlight the potential of HMC educational training programs to enhance competence among rehabilitation professionals so that the working methodology for SPHMM and person‐centered transfers can be more efficiently implemented to meet patient needs around the clock within interdisciplinary teams.

## Author Contributions

**Jeanette Melin:** conceptualization, formal analysis, methodology, project administration, validation, visualization, writing – original draft. **Nicola Parmelund:** data curation, funding acquisition, project administration, writing – review and editing. **Magnus Johansson:** software, supervision, validation, writing – review and editing.

## Conflicts of Interest

J.M. has received research support from HMC, and N.P. is a joint owner in HMC. Partial financial support was also received from RISE internal platform for Categorical Based Measures.

## Transparency Statement

The lead author, Jeanette Melin, affirms that this manuscript is an honest, accurate, and transparent account of the study being reported, that no important aspects of the study have been omitted, and that any discrepancies from the study as planned (and if relevant, registered) have been explained.

## Supporting information

Appendix.

## Data Availability

The data that support the findings of this study can be made available from the HMC Sweden upon reasonable request.
