# Supplementary material for: Rehabilitation Professionals' Self‐Perceived Competence in Safe Patient Handling and Mobility Methods Before and After Training: A Cohort Study
Source: Health Sci Rep. 2025 Apr 23;8(4):e70755. doi: 10.1002/hsr2.70755 (PMC12018280; doi:10.1002/hsr2.70755)
Supplement: Supplementary file 1 — Appendix. [file HSR2-8-e70755-s001.docx]

Appendix

On the projects OSF page, <https://osf.io/vmrkz/>, the four appendixes can be retrieved.

Appendix 1 provides codes and reports the Rasch analysis of self-perceived SPHMM among OT and PT, which is used for pre-post statistical analyses in Appendix 3.

<https://osf.io/vmrkz/files/osfstorage/6753de38c336bb94cb3c152c>

Appendix 2 provides codes and reports the Rasch analysis of self-perceived SPHMM utility among OT and PT, which is used for post analyses in Appendix 4.

<https://osf.io/vmrkz/files/osfstorage/6753de386cdeadeb10860fe7>

Appendix 3 provides codes and reports statistical analysis of self-perceived SPHMM among OT and PT before and after 3 months

<https://osf.io/vmrkz/files/osfstorage/6753de3851428063ff95e54a>

Appendix 4 provides codes and reports statistical analysis self-perceived SPHMM utility among OT and PT after 3 months

<https://osf.io/vmrkz/files/osfstorage/6753de386cdeadeb10860fe9>
